# Neurogenesis in the rat neonate's hippocampus with maternal short‐term REM sleep deprivation restores by royal jelly treatment

**DOI:** 10.1002/brb3.2423

**Published:** 2021-11-22

**Authors:** Atena Khodaverdiloo, Mona Farhadi, Melikasadat Jameie, Seyed behnamedin Jameie, Vahid Pirhajati

**Affiliations:** ^1^ Department of Microbiology Karaj Branch Islamic Azad University Karaj Iran; ^2^ Iranian Center of Neurological Research Tehran University of Medical Sciences Tehran Iran; ^3^ Neuroscience Research Center (NRC) Iran University of Medical Sciences Tehran Iran; ^4^ Department of Anatomy Iran University of Medical Sciences Tehran Iran

**Keywords:** brain‐derived neurotrophic factor, hippocampus, neurogenesis, rapid eye movement sleep deprivation, royal jelly

## Abstract

**Background:**

Numerous studies have shown the effects of rapid eye movement sleep deprivation (REM‐SD) on behavior and brain structures. The impact of REM‐SD on learning and memory, thus neurogenesis, has been reported in previous studies. Royal jelly (RJ) is known as the wealthiest biological nutrient with various physiological properties. This study aimed to study the possible effect of RJ on neurogenesis of the rat hippocampus neonates following exposure of mother to REM‐SD during pregnancy.

**Methods:**

Thirty neonate rats from 15 pregnant Wistar rats were used. To induce REM‐SD, the flowerpot method was used. The pregnant rats were divided into five groups (*n* = 3): group 1, no treatment; group 2, REM‐SD; groups 3, 4, and 5, REM‐SD +RJ. The former group received 72 h REM‐SD during pregnancy (days 7, 14, 21), and the latter group received REM‐SD + RJ (three trial groups). At week 4, the rat neonates of all groups were sacrificed (*n* = 6 each group). Their brains were fixed, removed, and prepared for Nissl and Hoechst 33342 staining. By using real time polymerase chain reaction methode the brain‐derived neurotrophic factor BDNF gene expression was studied (RT‐PCR), brain‐derived neurotrophic factor (BDNF) gene expression was studied. The results were analyzed statistically, and the *Pv*  < .05 was considered significant.

**Results:**

The results showed a significant decrease in the number of neurons in the hippocampus of neonatal rats of REM‐SD mothers compared to the neonates of the mother with REM‐SD + RJ. REM‐SD also led to an increase in apoptosis reaching the neonates from the REM‐SD + RJ animals. High expression of BDNF was observed in the hippocampus of the neonates from REM‐SD + RJ treated mothers.

**Conclusion:**

RJ acts as a neuroprotective agent that could compensate for the effects of REM‐SD on learning and memory via restoring neurogenesis.

## Graphical abstract

In order to study the effect of royal jelly (RJ) on neurogenesis following early exposure to rapid eye movement sleep deprivation (REM‐SD) in rat neonates, the present study designed to study the effect of Royal Jelly. We believe that RJ has a dual role via brain‐derived neurotrophic factor (BDNF) following REM‐SD; increasing the expression of BDNF in the hippocampus stimulates cell proliferation and differentiation and inhibits apoptosis.

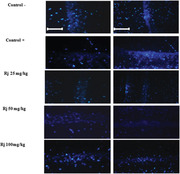

## 1 INTRODUCTION

Sleep is a universal phenomenon among all vertebrates, especially in higher‐order mammalians. Sleep follows a circadian rhythm that consists of two phases; rapid eye movement (REM) and non‐REM sleep. REM sleep is a unique physiological phase that occurs intermittently during sleep and is expressed in higher‐order mammals, including humans. Many researchers have shown the neuronal structures and circuits that are involved in REM sleep regulation. It is shown that many neurological, neurodegenerative diseases and several psychosomatic illnesses are associated with sleep disorders, mainly loss of REM sleep. Prolonged REM‐SD may lead to death. REM‐SD leads to many biochemical and cellular changes. It is shown that REM‐SD affects intracellular calcium, membrane permeability, and protein expression at the cellular and molecular levels, including enzymes, hormones, and neurotransmitters. From the research point of view, SD is accepted as an experimental method to investigate the neural bases of sleep phenomena.

Regarding this, the effects of REM‐SD on learning and memory tasks were reported by many researchers. The hippocampus is well known for its role in memory processing, consolidating short‐term memory information into long‐term memory, and neurogenesis (Tottenham & Sheridan, 2010). It is not clear exactly what effect REM‐SD has on the hippocampus structure and function. Animal experiments have been shown that REM‐SD leads to events which may reduce neurogenesis (Mostaghimi et al., 2005). Mueller et al. (2008) reported a significant decrease in neurogenesis in the adult rat hippocampus following REM‐SD. Junek et al. (2010) showed that long‐term SD reduced cell proliferation and hippocampal neurogenesis in adult rats, whereas short‐term SD had no such effect. Accordingly, they claimed that rats with short‐term SD, with an unknown possible compensatory mechanism, produced more precursor hippocampus cells by accelerating the cell cycle and this demonstrated the importance of using multiple cell cycle markers for neurogenesis in the hippocampus (Junek et al., 2010). Hajali et al. (2012) studied the effects of REM‐SD on short‐term memory, spatial learning, and anxiety‐like behaviors in male and ovariectomized (OVX) female rats. Their results showed that short‐term REM‐SD caused significant impairment in learning, function, and spatial memory of the OVX female rats, but not in the male rats (Hajali et al., 2012). Naseri et al. (2014) showed the effects of REM‐SD on neurogenesis, learning, and memory of the rats with REM‐SD. They confirmed the dramatic effects of REM‐SD on neurogenesis (Naseri et al., 2014). Spano et al. (2019) reported the synaptic changes in the CA1 region of adult mice hippocampus by rearranging the sleep‐dependent synaptic structure. Therefore, the role of sleep in maintaining synaptic plasticity may be related to the hippocampus, an important area for synaptic learning and neuroplasticity (Spano et al., 2019). Alquraan et al. (2019) studied the effect of omega‐3 on memory impairment induced by long‐term SD and found that the mice with SD showed significant errors in short‐ and long‐term memory tests compared to controls. Jameie et al. (2019) reported the positive effects of exogenous melatonin on locus coeruleus of the adult rat following REM‐SD; they showed that the exogenous melatonin acts as a neuroprotective agent to compensate the effects of REM‐SD. Godinez et al. (2019) showed that melatonin administration to animals with SD increased MECP2 gene expression and decreased SIRT1 gene expression in the dentate gyrus. They also observed that let‐7b, mir‐132, and mir‐124 in this region increased after melatonin administration, but did not increase in sleep deprivation. They found that melatonin could epigenetically increase cell growth in this brain area in sleep‐deprived animals (Hinojosa‐Godinez et al., 2019). Brain‐derived neurotrophic factor (BDNF) is a well‐known neurotrophin in the brain that plays important roles in neural development, neural survival, neurogenesis, learning, memory, appetite, sleep, and neuroplasticity the serotonergic, noradrenergic, and dopaminergic neural circuits (Hassanzadeh et al., 2021). In the last few years, it has been hypothesized that BDNF level is related to depression, sleep, and insomnia. Studies show that BDNF plays an essential role in sleep regulation, and any decrease in serum level of BDNF is directly related to insomnia (Faraguna et al., 2008). It is shown that 12 h of SD increases the expression of the BDNF in the hippocampus and increases the activity of PI3K/AKT/GSK3β/mTOR and TrkB/CREB/ERK signaling pathways (Yan et al., 2016). The neurotrophin hypothesis for BDNF is formed based on the fact that any stress‐related mental disorders such as sleep disturbances result from stress‐induced decreases in BDNF expression. Thus, we hypothesized that REM‐SD could lead to dramatic changes in BDNF expression in the hippocampus.

Royal jelly (Rashidy‐Pour, 2019), a secretion of honeybee hypopharyngeal and mandibular glands, is a nutrient consisting of water, various protein, monosaccharide, fatty acid, and hydroxy‐2‐decanoic acid. Its main proteins (MRJPs) consist of a family of nine proteins from MRJP1 (called royalactin), MRJP2, MRJP3, MRJP4, and MRJP5 in a gel secreted by worker bees, of which MRJP1 is the most abundant one. These five proteins contain 83% to 90% of the total royal jelly proteins (Buttstedt et al., 2014). The protective, anti‐aging, neuromodulatory, and anti‐cancer effects of royal jelly (RJ) have been reported in various studies (Lirdi et al., 2008). You et al. (2019) showed the effectiveness of RJ on improving learning and memory tasks in animal models of Alzheimer's disease. They showed that RJ inhibits neuronal apoptosis by activating the cAMP/PKA/CREB/BDNF pathway (13) since many articles have demonstrated that REM‐SD can affect neurogenesis and consequently learning and memory as well as the biological properties of RJ. We hypothesized that the RJ might have unproven neurogenesis effects in the hippocampus. Accordingly, we decided to investigate the effect of RJ on neurogenesis, apoptosis, and BDNF gene expression in the hippocampus of the rat neonates with maternal short REM‐SD.

## 2 MATERIAL AND METHOD

### 2.1 Reagents

Royal jelly was purchased from Shahdineh Gol (Iran), Hoechst3334, and cDNA synthesis kit—Gene All—from Sigma‐Aldrich Co.

### 2.2 Biological model and groups

In this study, 30 rat neonates from pregnant Wistar rats were used. The animals were purchased from the Animal Care Center of Iran University of Medical Sciences (IUMS). During the experiment, the pregnant rats during pregnancy had free access to water and food and were kept under standard dark‐light cycle 12:12, room temperature, and humidity. Animal Ethical Committee of NRC/IUMS approved all procedures used in this study. After pregnancy confirmation by observing vaginal plug, the 15 pregnant rats were randomly divided into five study groups (*n* = 3): group 1; the pregnant animals with no intervention (intact, negative control group), group 2; the pregnant animals received 72 h of REM‐SD (positive control group), and group 3, 4, and 5; the pregnant animals that received the same REM‐SD and different doses of RJ (25, 50, 100 mg/kg of RJ considered as the three trial groups, Exp1, Exp2, and Exp3). Animals of the first group had no intervention and were kept in normal conditions, and their neonates were considered as negative controls. The second group received 72 h REM‐SD during pregnancy (days 7, 14, 21), and their kids were the positive control group, and the last groups received REM‐SD (days 7, 14, 21) + RJ (25, 50, 100 mg/kg) and their neonates were the trial animals. One week after delivery, the rat neonates of all groups, as the study groups, were sacrificed and prepared for subsequent studies. To study neuronal density and cell count, apoptosis, and expression of BDNF, Nissl staining, Hoechst staining, and real time polymerase chain reaction methode the brain‐derived neurotrophic factor BDNF gene expression was studied (RT‐PCR) were respectively used.

### 2.3 Induction of REM‐SD

To induce REM‐SD, the disk over water or flowerpot technique was used (Ashraf et al., 2000). The apparatus used in this technique is a cubic Plexiglas cage (30 × 30 × 30 cm) with a bar 10 cm high in the middle and plates with different diameters of 5 to 12 cm. To reduce the stress of immobility, plates with diameters of 12, 10, and 7 cm were prepared. The apparatus was filled with fresh water for a depth of 1 cm below the plate that the animals were put on. Three days before the beginning of the 72‐h RSD period, the rats were placed on plates of 12, 10, and 7 cm for 3 h, respectively, for adaptation. During the RSD period, the rats had access to fresh water and adequate food ad libitum. The apparatus was placed in an isolated place with room temperature, enough humidity, no noise, and dark‐light cycle of 14–10 h. The animal could not leave the apparatus during REM‐SD. Sleep deprivation of 24 h was performed three times in 7, 14, and 21 prenatal days of pregnant rats. Between the days of REM‐SD, the animals were transferred into a regular single cage. One week after delivery (7 PN day), the rat neonates were prepared for the study (Table 1). All the procedures used in this study were approved by the ethic committee of Neuroscience Research Center of Iran University of Medical Sciences (ethical code IR.IUMS.REC.1398.1214).

**TABLE 1:** Study groups including the neonates’ pregnant rats. The induction of REM‐SD was done for 72 h at 7, 14, and 21 days of pregnancy for all pregnant rats. REM‐SD for all groups started at 10:00 a.m. and continued for 24 h, repeated three times at the mentioned days (total hours of REM‐SD = 72 h/pregnancy) . RJ treatment was done in three doses 2 h before each time of REM‐SD

Group treat	**REM‐SD72** **h**	RJ 25 mg/kg	RJ 75 mg/kg	RJ 100 mg/kg	*n*Neonates
Intact neg cont	**×**	**×**	**×**	**×**	**6**
Positive cont	**√**	**×**	**×**	**×**	**6**
Exp1	**√**	**√**	**×**	**×**	**6**
Exp2	**√**	**×**	**√**	**×**	**6**
Exp3	**√**	**×**	**×**	**√**	**6**

### 2.4 Treatment with RJ

The REM‐SD + RJ group animals received RJ soluble in distilled water intraperitoneally in three doses of 25, 50, 100 mg/kg 2 h before REM‐SD in 7, 14, and 21 days.

### 2.5 Nissl staining

After delivery and at week 4, the neonate rats were sacrificed by a lethal dose of anesthetic agents. Animals were anesthetized with an intraperitoneal injection of ketamine–xylazine (80 mg/kg ketamine‐10 mg/kg xylazine) mixture.

Immediately, the animals were perfused and fixed by aldehyde solutions transcardially. Their brain was removed and kept in graded sucrose solution as the cryoprotective agent, by using a freezing microtome (Leica 3000 Germany) and coronal sections of 10 μm were prepared. Nissl staining was done as mentioned in our previous studies (Hassanzadeh et al., 2018; Jameie et al., 2021). By using Image J software, the neuronal number and density of the hippocampus number were studied.

### 2.6 Hoechst staining

To study apoptotic neurons, Hoechst staining was used. Hoechst3334 stains chromatin and makes it visible under a fluorescent microscope. For this purpose, the assigned neonates for this staining were sacrificed immediately; their brains were removed in fixed in aldehyde solution in PBS for 30 min at room temperature. After that, the brains were washed several times with PBS and then incubated with 10 μl/ml Hoechst dye at room temperature again for 30 min. After washing, the cells were viewed and counted at 360 nm with a fluorescent microscope (Manouchehrabadi et al., 2020).

### 2.7 Real‐time PCR

To study BDNF gene expression, the rat neonates’ sacrificed brains were separated. The tissues were immediately cleaned and preserved at −80°C, then homogenized and lysed (No. 305‐101 Made in South Korea). All works are performed under a laminar hood and on ice. Quantitative analysis of the extracted RNA using adsorption was performed at OD 260/280 by nanodrop. To make complementary DNA molecules, cDNA synthesis kit—Gene All—Cat. No. 601‐005 made in South Korea were used. The ingredients were first combined in a tube according to the kit instructions. For this purpose, 2 μg of sample RNA was mixed with 1 μl of random hexamer primer, 1 μl of dNTPs, and RNAse‐free water was added until the total volume reached 14 μl. The materials were centrifuged inside the tube for a short time to ensure that they were mixed. They were then incubated at 65°C for 1 min. To measure the expression levels of BDNF mRNA, the quantitative Real time‐PCR method was used using Premix SYBR Green II. The reaction mixture at final volume was 20 μl. Primers were designed based on BDNF and GAPDH genes in NBCI Gene Bank as Housekeeping gene was considered (Table 2). The temperature program used in real time‐PCR includes 95°C for 10 min, 95°C for 15 s, and 60°C for 1 min (40 cycles repetition). Cycle threshold (Ct) values were obtained through the auto Ct function. Following efficiency correction, the mean CT value was calculated and then normalized to the reference using delta (D) CT.

**TABLE 2:** Primer sequence for quantitative real‐time polymerase chain reaction

Primer name	Primer sequence
BDNF	F‐AGTGATGACCATCCTTTTCCTTAC
BDNF	R‐CCTCAAATGTGTCATCCAAGGA
GAPDH	F‐ACTCCACTCACGGCAAATTC
GAPDH	R‐TCTCCATGGTGGTGAAGACA

### 2.8 Statistical analysis

The results were analyzed by SPSS (version 21) statistical software using One Way ANOVA and Tukey test, the *Pv*  < .05 considered significant. The normality of the data was tested by the Kolmogorov–Smirnov exam.

## 3 RESULTS

### 3.1 Results of Nissl staining of hippocampus tissue

RJ restore the number of living cells in the hippocampus of rat neonates with maternal REM‐SD.

**FIGURE 1:**
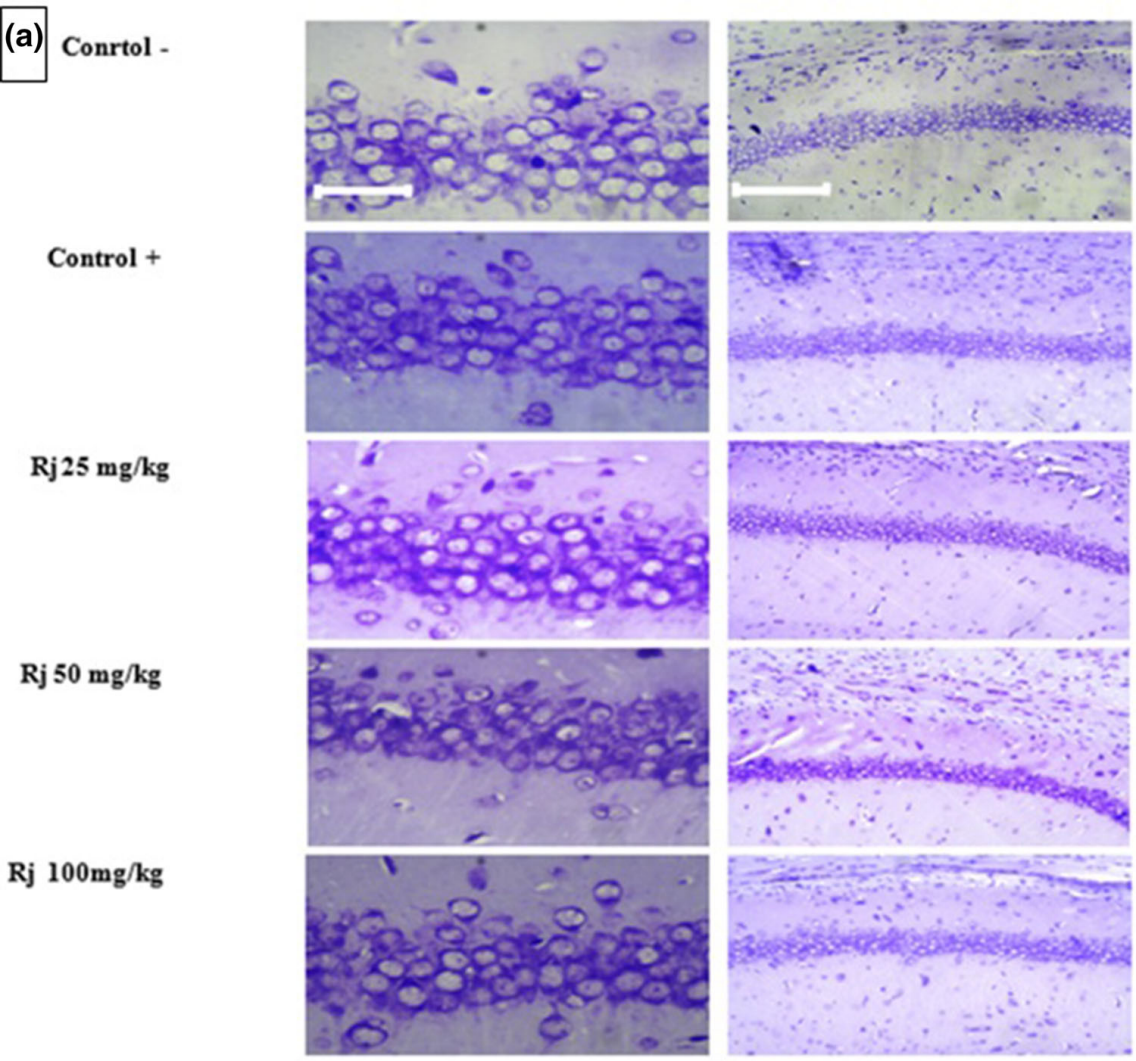
(a) Represents the Nissl staining of the number of live cells with the different doses of royal jelly on negative, positive control, and EXP groups. 10× and 40× magnification. (b) Graph of the number of live neurons in different groups. *Significant with the defect amount with a positive control group. #Significant with the negative control group

Nissl staining was performed to evaluate the number of living cells in the hippocampus. The data showed that the number of cells in the positive control group (REM‐SD) was significantly lower than the negative control group. In the neonates whose mother received RJ at doses of 25, 50, and 100 mg/kg, the number of neurons was significantly higher than the positive group (REM‐SD) animals that showed the possible protective role of RJ. Our data also showed that the effect of RJ was dose‐dependent, that is, the higher dose of RJ led to the high number of lived neurons (*p* < .001) (Figure 1a,b).

### 3.2 Results of Hoechst3334 staining in the hippocampus

Apoptosis significantly decreased in the hippocampus of rat neonates with maternal REM‐SD.

**FIGURE 2:**
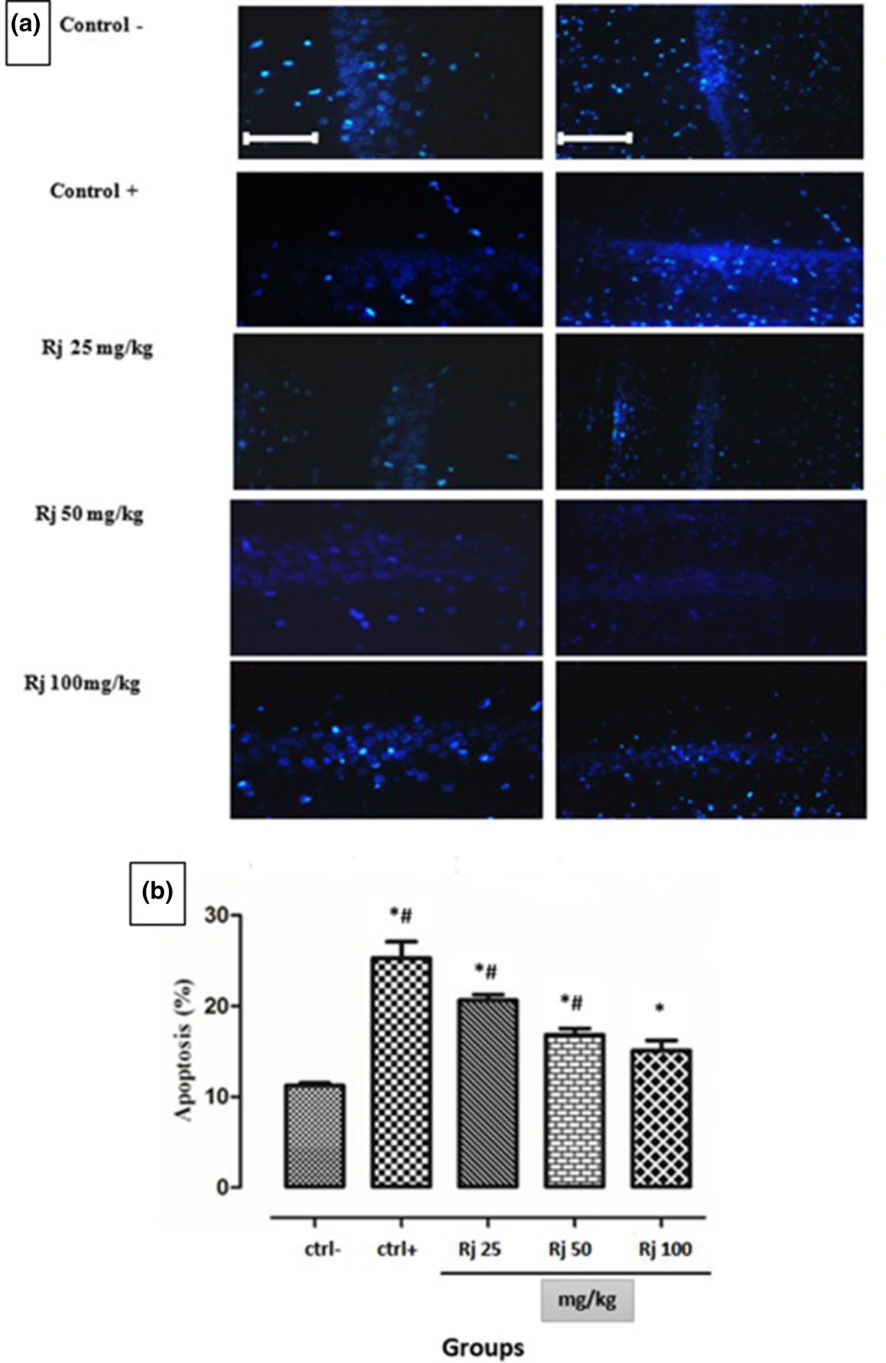
(a) Number of apoptotic cells in the hippocampus in different groups. Royal jelly. (b) Graph of apoptotic percentage at doses of 25, 50, and 100 mg/kg caused a significant reduction (*p* < .001) in the number of dead cells in the hippocampus of the animals with mother REM‐SD induced

Hoechst3334 staining was used to detect apoptotic cells in the hippocampus of different groups. Our data showed that the number of apoptotic cells in the negative control group was significantly less than the positive control group, that is, neonates from REM‐SD mothers. Comparing the results between the neonates from REM‐SD animals with mothers who received different doses of RJ showed higher apoptotic cells in the neonates of the REM + SD group. Royal jelly caused a significant decrease (*p* < .001) of apoptotic cells in the hippocampus of the neonates from mothers with REM‐SD +RJ compared to the positive control group (REM‐SD). The effect of royal jelly was dose‐dependent at doses of 25, 50, and 100 mg/kg (Figure 2).

### 3.3 Results of BDNF gene expression

RJ treatment led to increased BDNF expression in the hippocampus of rat neonates with maternal REM‐SD.

**FIGURE 3:**
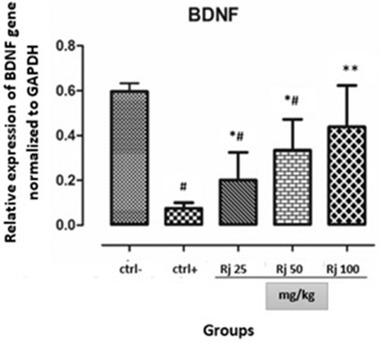
Relative expression of BDNF gene normalized to GAPDH expression in different groups. ^*^Significant with the positive control group. ^#^Significant with the negative control group. ^*^ *Pv* < .05, ^**^ *Pv* < .01

Real‐time PCR data (Figure 3) showed that the BDNF expression in hippocampal significantly decreased in the neonates of the positive control group (REM‐SD) compared to neonates from harmful control animals (*p* < .0001). Administration of royal jelly with different doses causes a significant increase (*p *< .001) in BDNF expression in the hippocampus of the neonates of the REM‐SD + RJ mothers compared to neonates from the positive control group (REM‐SD). All doses of RJ led to a significant increase in BDNF expression in a dose‐dependent manner.

## 4 DISCUSSION

In this study, the effect of royal jelly was investigated on neuronal survival, apoptosis, and the BDNF expression in the hippocampus of the rat neonates with maternal short‐term REM‐SD. Our data at first showed that REM‐SD caused apoptosis and a decrease in BDNF expression in the hippocampus. Much research has been done since Klitman and Aserinsky first identified rapid eye movement sleep (REMS) in 1953. Since then, REM‐SD is supposed as a research strategy to study the neural bases of sleep. Many researchers have shown the structures involved in REM sleep regulation and the effects of REM‐SD on certain structures of the brain. Many reports show the REM‐SD at different times, either short or long term, has dramatic cellular and molecular effects on brain structures. Sakai (2015) reported the effects of REM‐SD on the neurons of laterodorsal tegmentum pontine in mice. By this research, he showed the importance of this area in REM‐sleep regulation (Sakai, 2015). Kreutzmann et al. (2015) showed the effects of sleep deprivation on hippocampal neuronal plasticity and cognitive function. Based on their findings, they suggest that SD impairs hippocampal neuroplasticity, learning, and memory process by attenuating intracellular cyclic adenosine monophosphate ‐protein kinase A (PKA) signaling, which may lead to alterations in cAMP response element‐binding protein (CREB)‐mediated gene transcription, neurotrophic signaling, and glutamate receptor expression (Lirdi et al., 2008). They claimed that in long‐term or chronic SD conditions, a reduction of hippocampal cell proliferation, and neurogenesis occurs, which may eventually lead to the reduced hippocampal volume that finally destroys hippocampal neuroplasticity function contributing to cognitive and psychiatric disorders (Kreutzmann et al., 2015). Although the molecular mechanisms underlying sleep and sleep deprivation remain to be investigated, available pieces of evidence suggest that SD may act via neuronal apoptosis in certain nuclei of the brain. The effect of REM‐SD on cytomorphometeric parameters of dorsal raphe nucleus in rat brain reported by Ranjan et al. (2010). They claimed the changes in DRN following REM‐SD is mediated by noradrenalin mechanism (Ranjan et al., 2010). In another research in 2008 by Mueller et al. (2008), the effect of REM‐SD on hippocampal neurogenesis fowling adrenal stress hormone reported. They showed that the inhibition of neurogenesis in the hippocampus occurs independently from adrenal stress hormones that may suggest other mechanisms for inhibiting neurogenesis. In another similar research by Guzman‐Marin et al. (2007), the reduction of neurogenesis in the dentate gyrus of the hippocampus following REM‐SD was reported. They believed that the elevated glucocorticoids do not account for the reduction in cell proliferation induced by the SD, although a small contribution of stress is not excluded (Guzman‐Marin et al., 2007). Junek et al. (2010) showed that short‐term REM‐SD (12 h) altered the dynamics of hippocampal cell proliferation in adult rats. Their findings claimed different effects that short‐term (12 h) SD transiently produces more hippocampal progenitor cells via cell cycle acceleration (Junek et al., 2010). Putting these researches together, there might be various mechanisms that influence the neurogenesis in the hippocampus following SD. Numakawa et al. (2017) showed the importance of adequate neurogenesis in mental health and brain disorders. Their research reported that the neurotrophic factors are well known for positive effects on the cells' proliferation and differentiation in embryos and adults. The BDNF has been extensively studied among these neurotrophic factors because of its important role in proliferation and differentiation. It is generally accepted that neurogenesis is restricted to the subventricular zone (SVZ) of the lateral ventricles and subgranular zone (SGZ) in the hippocampal dentate gyrus. The neurogenesis in these is not limited to embryonic life but continues in adults (Numakawa et al., 2017). One of the factors that influence the process of neurogenesis is BDNF. It is shown that the expression of BDNF in the hippocampus and cortex of Rhesus monkey is higher than in another area of the brain, notably the expression of BDNF reaches its high level during the neurodevelopmental period (Mori et al., 2004). In addition, increased neurogenesis in the granule cell layer after BDNF infusion into the hippocampus of adult rats was reported (Scharfman et al., 2005). Bath et al. (2012) showed that BDNF is supposed to be the strong candidate molecule regulating adult neurogenesis. How SD leads to a decrease in neurogenesis remains a question. Some studies have demonstrated functional interactions between neurotrophic and glucocorticoid (GCs) in neural events, such as neurogenesis. Under certain stress like SD, the level of GCs increases that may lead to decreased BDNF expression, subsequently causes inhibition of neurogenesis. Our research showed decreased BDNF expression and a subsequent decrease in neurogenesis in neonates from mothers with REM‐SD in line with other researches. Although, we did not study the level of GCs following REM‐SD, it seems the interaction between GCs and BDNF plays an important role in this phenomenon.

Using RJ in REM‐SD + RJ, the level of BDNF expression increased, and the number of apoptotic cells significantly decreased. The question is how RJ could insert this effect. Many studies have examined the impact of specific protective agents on the impact of REM‐SD.

Alquraan et al. (2019) studied the effect of omega‐3 on memory deficits following chronic REM‐SD and found that omega‐3 could improve memory deficit in animals with REM‐SD. Omega‐3 administration also inhibited the reduction of glutathione peroxidase, catalase, and GSH/GSSG ratio in the hippocampus (Alquraan et al., 2019). Omega‐3 acts as an antioxidant and could improve cellular conditions following certain stress to neurons. Although it is not reported whether RJ has omega‐3 or not, it has antioxidant properties with possible same mechanism as omega‐3. Hinojosa‐Godinez showed that melatonin administration following SD increased MECP2 gene expression and decreased SIRT1 gene expression in the dentate gyrus. They also observed that the expression of let‐7b, mir‐132, and mir‐124 microRNAs in this region increased after melatonin administration, but did not increase with sleep deprivation. They found that melatonin could epigenetically increase cell proliferation and differentiation in the hippocampus of the animals with SD (Hinojosa‐Godinez et al., 2019). Novati et al. (2011) reported that the volume of the dorsal hippocampus decreased by 10% in in adult rats with chronic REM‐SD. According to their results, the reduction in hippocampal size was not associated with significant changes in the number of newly produced BrdU‐labeled cells or alterations in Doublecortin (DCX) expression as a marker of immature neurons (Novati et al., 2011), which is different from the results of our study. It seems that the type of sleep deprivation can be effective in this regard. Saadati et al. (2014) studied the effects of regular treadmill exercise and sleep deprivation on expression levels of BDNF expression in the hippocampus of OVX rats. They showed that the BDNF expression decreased significantly following 72 h of REM‐SD, and regular treadmill exercises in animals with SD led to a significant increase in hippocampal BDNF expression (Saadati et al., 2014).

Regarding the action mechanism of RJ on apoptosis and subsequent cell proliferation and differentiation, You et al. showed RJ could inhibit apoptosis via activating the cAMP/PKA/CREB/BDNF pathway in an AD mouse model (Mueller, 2008). Accordingly, we think that RJ has a dual role via BDNF following REM‐SD; increasing the expression of BDNF in the hippocampus stimulates cell proliferation and differentiation and inhibits apoptosis via the cAMP/PKA/CREB/BDNF pathway at the same time.

## 5 CONCLUSION

Based on our findings, RJ acts as a neuroprotective agent that could compensate for the effects of REM‐SD on learning and memory via restoring neurogenesis. Although we know that the exact mechanisms of RJ action on neurogenesis and subsequent learning and memory needs more research, we believe that RJ could be a promising medicine for certain neurodegenerative and neurological disorders such as AD with sleep disturbances origin. Easy access to royal jelly and its low cost are important advantages that reduce the cost of treating some neurodegenerative diseases. Accordingly, we suggest strongly for more research on the effects of RJ and its action mechanisms on different neurodegenerative diseases.

## CONFLICT OF INTEREST

The authors declare no conflict of interest.

## AUTHOR CONTRIBUTIONS

M. Farhadi, S. B. Jameie, and V. Pirhajati: Study design; budget planning; methodology. A. Khodaverdiloo and Jameie Melikasadat: Methodology; methods and microscopic study and reports. S. B. Jameie and M. Farhadi: Manuscript preparation. Jameie Melikasadat and S. B. Jameie: Editing of the manuscript. Jameie Melikasadat: Data analysis.

### PEER REVIEW

The peer review history for this article is available at https://publons.com/publon/10.1002/brb3.2423

## Data Availability

Please contact the author for data requests.
